# Developing a Tumor Microenvironment in Rotating Human Melanoma Cell Cultures: Study of a Novel Preclinical Model

**DOI:** 10.1021/acsomega.5c02682

**Published:** 2025-06-20

**Authors:** Kamil Wawrowicz, Martyna Durak-Kozica, Mateusz Wierzbicki, Ewa Ł. Stępień

**Affiliations:** † Department of Medical Physics, M. Smoluchowski Institute of Physics, Faculty of Physics, Astronomy and Applied Computer Science, Jagiellonian University, Kraków 31-007, Poland; ‡ Center for Theranostics, Jagiellonian University, Kraków 31-007, Poland; § Department of Nanobiotechnology, Institute of Biology, 49561 Warsaw University of Life Sciences, Warsaw 31-007, Poland

## Abstract

The 3D culture is currently the most promising technique used in preclinical studies for drug testing. One of the main challenges in preclinical *in vitro* studies is the long-term observations (over 21 days), which can be performed using cell spheroids maintained in bioreactor conditions—tumorspheres. Here, our goal was to develop and characterize two types of human melanoma cell lines: primary (FM55p) and malignant (WM266-4) cultures, in rotating bioreactors as an alternative to *in vitro* modeling. We proposed potential end points of evaluation: viability tests (PI–propidium iodide imaging of necrotic core, PI/annexin V flow cytometry, trypan blue staining) and modulators of the epithelial-mesenchymal transition pathway (spectral flow cytometry of vimentin, MCAM, and CD44 expression). In addition, with confocal microscopy, we visualized regulators of angiogenesis (VEGF-β) and single-cell spectral flow cytometry (E- and VE-cadherin). The tumorspheres showed the ability to grow for at least 1 month to reach millimeter sizes. Their morphology was improved compared to traditional 3D spheroids, and we observed changes in the tumor microenvironment and the expression of key proteins. Our study demonstrated the utility of tumorspheres in personalized medicine and drug development The advantage of the described models is the creation of prospects for further development of maintaining cell models that are hybrid systems combining the features of spheroids and organoids for preclinical and translational research.

## Graphical abstract

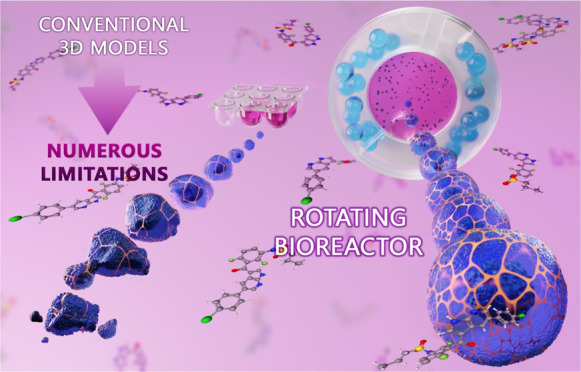

## 1 Introduction

Significant differences at various cellular levels between *in vitro*, *in vivo*, and clinical tumor models are one of the major challenges hindering the progress in cancer diagnosis and therapy. *In vitro* assessment, as a preliminary evaluation step, has a critical impact reflecting further stages’ success rates as they are used not only for *in vitro* evaluation but are subsequently applied for tumor inoculation in an animal model.^1–3^

The applicability range of traditional cell cultures enables only basic research such as drug uptake or cell survival. Two-dimensional growth in an unnatural flattened shape impairs the cell morphology, while uniform distribution of nutrients and medium results in high homogeneity. Synchronized cell cycles contribute to changes in the cellular response and growth, leading to abnormal outcomes. Furthermore, cell-to-cell interactions, limited to the boundary of the cell outline, lead to changes in genes and protein expression, also affecting intercellular signaling and more importantly the development of the tumor microenvironment (TME). Consequently, these models, due to numerous morphological and physiological shortcomings, do not effectively assemble the clinical tissues.^4^

Introducing a spatial, three-dimensional (3D) cell culture model, classified as spheroids (cell culture-derived) or organoids (tissue-derived), offers advantages over the traditional ones.^5^ This environment permits cells to preserve or restore their inherent morphological characteristics, thereby facilitating the establishment of a sophisticated cancer microenvironment (milieu) exhibiting a higher degree of complexity, relative to its two-dimensional (2D) counterpart. More pronounced cell differentiation coupled with improved intercellular interactions via ions, small molecules, and electrical signals makes them more suitable for simulating tumor tissue.^6^ The expression of genes and proteins can be similar to *in vivo* conditions^7^ and may allow the introduction of tumor growth factors, such as gravity and mechanical stimulation, which are unavailable in 2D cultures.^8,9^ The main disadvantage of typical 3D cell cultures is the limited culture time (∼several days) and poor model reproducibility, having a primary importance for the extensive preclinical evaluation various types of novel therapeutic approaches.^10,11^ Additionally, reduced nutrients and oxygen penetration and insufficient elimination of waste and metabolic products are other limitations of typically used methods. These features as well as economic aspects do not offer encouraging benefits that could support their wide implementation into preclinical protocols.^12^

One of the major requirements that could lead to improving the effectiveness of drug discovery is a holistic approach. Redefining model types and their use must be the first step toward improving the quality of preclinical evaluation. Furthermore, recent FDA regulations also force the replacement of *in vivo* models with advanced 3D cell cultures,^13^ emphasizing the importance of rapid progress in such systems. Consequently, there is an urgent need to develop and characterize highly advanced 3D models for preclinical research, upgrading *in vitro* models or replacing animals. Addressing those needs, with this study, we aimed for developing and characterizing human melanoma tumorspheres cultured with rotating bioreactors as a promising alternative for *in vitro* tumor modeling (Figure 1). We proposed potential end points of evaluation: viability tests (PI—propidium iodide imaging of necrotic core, PI/Annexin V flow cytometry, trypan blue staining), and modulators of the epithelial-mesenchymal transition (EMT) pathway (spectral flow cytometry of vimentin, MCAM and CD44 expression). In addition, we visualized with confocal microscopy regulators of angiogenesis vascular endothelial growth factor type β (VEGF-β) and single-cell spectral flow cytometry (E- and VE-cadherin).

**Figure 1:**
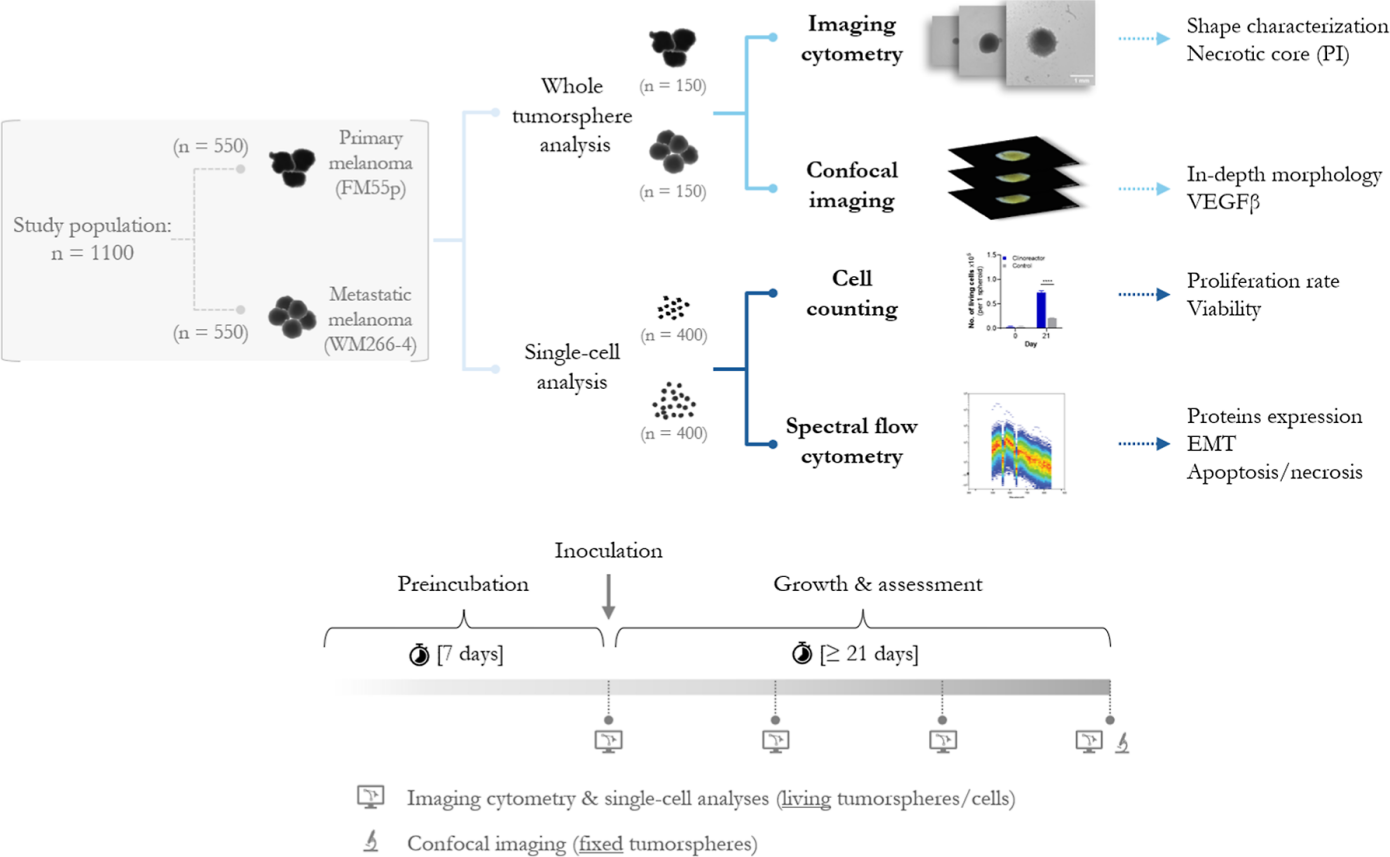
Study design and outline.

## 2 Materials and Methods

### 2.1 Cell Lines

Two human melanoma cell lines were used—primary (FM55p) and malignant (WM266-4) purchased from ESTDAB Melanoma Cell Bank (Tübingen, Germany) and cultured according to the manufacturer recommendations as we previously described.^14^

### 2.2 Spheroids’ Preincubation

Seven days before the experiment, FM55p and WM266-4 cells (10^3^ cells in 250 μL/per well) were seeded into 96-well *U*-bottom SPL3D Cell Floater plates (SPL Life Sciences Co., Ltd., Pocheon-si, South Korea). The goal of this step was to ensure better model reproducibility of the models by precultivating the cells until they initially form spheroids before transferring to the bioreactor. During this initial period of incubation (37 °C, 5% CO_2_), medium renewal was performed every 2 days. Structures cultured in bioreactors are further referred to as tumorspheres, while plate-based models are further referred to as spheroids.

### 2.3 Large-Sized Spheroids Culturing

The culture of large-sized spheroids was performed using the ClinoStar (CelVivo, Chevy Chase, MD, USA) system designed for generating *in vitro* models under conditions resembling the tumor environment in a living organism. For this purpose, 1 day before the transferring of spheroids, the bioreactor chamber was equilibrated with 25 mL of sterile water (4 °C, overnight) to hydrate the moisture beads. Then, the cell culture chamber was washed twice with complete medium. Finally, the chamber was filled with ∼6 mL of culture medium and placed in the ClinoStar incubator (37 °C, 5% CO_2_) for at least 2 h with gentle rotation (15 rpm). All procedures involving injection, replacement, and rising of the medium were performed using a 20 mL syringe with a needle to minimize the risk of contamination. After equilibration, the medium was removed, and 5 mL of fresh complete medium was added. Afterward, 75–96 spheroids (per one bioreactor) were inoculated. The cell chamber was filled with 10 mL of the culture medium. During the experiment, the rotation speed ranged from 15 to 35 rpm, and the adjustment depended on the size of the tumorspheres separately for each bioreactor. The day after the spheroid transfer, all aggregates were removed from the chamber to maintain homogeneity and optimal growing conditions. The medium was replaced every 2 days, and the bioreactor every 14 days, according to the manufacturer’s recommendations. Control spheroids (75–96 per group), growing in the 96-well *U*-bottom plates, were also transferred to new plates after day 14 and subjected to medium replacement every 2 days.

### 2.4 Spheroids Imaging—Morphology

Each week, starting on day 0, growth, shape, and physiological activity were evaluated. For this purpose, a Celigo bright-field (BF) and fluorescence imaging cytometer (Nexcelom Biosciences, Lawrence, MA, USA) was used. The morphology of spheroids and tumorspheres was quantified according to the mathematical formulas used in the software, and the doubling time was estimated accordingly (Supporting Information eq S1 and Table S1). Prior to each imaging, spheroids were transferred to the SPL3D Cell Floater plates and washed twice with culture medium to remove any cell fragments and debris. The acquisition setup was adjusted to the cell line type, shape, and size of the spheroid, and included prefiltering to determine analysis parameters such as colony diameter, tumorsphere area, and tumorsphere intensity range to exclude artifacts.

### 2.5 Necrotic Core Staining

PI staining of the necrotic core (PI, Merck & Co., Inc., Kenilworth, NJ, USA) is one of the most widely used techniques to visualize necrotic cells located deep in the spheroid structure.^15^ For this purpose, the spheroids were stained with a 0.5 mg/mL PI solution in Mg^2+^/Ca^2+^-free PBS. After transfer to a 96-well *U*-bottom plate and complete removal of the medium, the spheroids were placed in 50 μL of PBS, and then 50 μL of PI solution was added. After 30 min, the spheroids were washed three times with PBS and finally placed in 100 μL of PBS. Fluorescence imaging was performed using a Celigo cytometer, with a prefiltering step as described above. The resulting images were analyzed via Celigo version 5.3.0.0 and FIJI software.

### 2.6 In-Depth Tumorsphere Analysis with Confocal Imaging

For postexperimental sectioning, spheroids were fixed in 2.5% of glutaraldehyde (ThermoFisher Scientific) and snap frozen with liquid nitrogen in Tissue Freezing Medium (Leica Microsystems, Wetzlar, Germany). The spheroids were then cut into 5 μm thick sections in the CM 1900 cryostat (CM 1900; Leica Microsystems) at −20 °C. Sections were mounted on polylysine-coated coverslips and examined using a CKX 41 Olympus inverted phase-contrast microscope (Olympus, Tokyo, Japan).

Entire spheroid imaging was achieved by immunolabeling and clearing using CytoVista 3D culture Clearing kit (ThermoFisher Scientific, Waltham, MA, USA). Fixed spheroids were permeabilized in increasing concentrations of methanol at 4 °C: 50% methanol in PBS, 80% methanol in deionized water, 100% methanol. Subsequently, they were washed in 20% DMSO/methanol, then in 80% MeOH/H_2_O; in 50% MeOH/PBS; 100% PBS, and finally in PBS with 2% Triton X-100. Samples were incubated with CytoVista Antibody Penetration Buffer for 30 min and blocked in CytoVista Blocking Buffer for 24 h at 4 °C. Spheroids were incubated with a primary antibody diluted in CytoVista Antibody Dilution Buffer (VEGF-β Monoclonal Antibody, ThermoFisher Scientific) for 24 h at 4 °C. The spheroids were then washed and incubated with Goat anti-Mouse IgG (H + L) Cross-Adsorbed Secondary Antibody, Alexa Fluor 594 (ThermoFisher Scientific) for 24 h at 4 °C. Cell nuclei were counterstained with Hoechst 33342 (ThermoFisher Scientific), washed and dehydrated with increasing concentrations of methanol (50% MeOH/PBS; 80% MeOH/H_2_O; 100% MeOH). After methanol removal, the spheroids were placed in an 8-well chambered μ-Slide 8 coverslip (Ibidi, Gräfelfing, Germany) and cleared with a 3D Cell Culture Clearing Reagent. *z*-Stacks of spheroids were obtained with an FV-1000 confocal microscope (Olympus Corporation, Tokyo, Japan) and reconstructed with FIJI software.

### 2.7 Single-Cell Tumorspheres Analysis with Spectral Flow Cytometry

Between 25 and 50 tumorspheres per group were collected and precipitated. Subsequently, growing medium was removed and 200 μL of Accutase (Merck & Co., Inc., Kenilworth, NJ, USA) was added to ensure structures disintegration (5 min, 37 °C). After complete cell detachment, 800 μL of complete growing medium was added and samples were centrifuged (250*g*, 5 min). Cells were reconstituted in 1 mL of PBS/5% fetal bovine serum (FBS) and a 30 μL sample was collected for cell counting and trypan blue exclusion staining and counted with a Luna-II Automated Cell Counter (Logos Biosystems, Dongan-gu Anyang-si, Gyeonggi-do 14055 South Korea).

For spectral flow cytometry, following antibodies from BioLegend (San Diego, CA, USA) were used: VE-cadherin (PE antihuman CD144, clone BV9), E-cadherin (PE/Dazzle594 antimouse/human CD324, clone DECMA-1, isotype: Rat IgG1, κ), CD44 (BV711 antimouse/human, clone IM7, isotype: Rat IgG2b, κ), Vimentin (AF647 anti-Vimetin, clone W16220A, isotype Rat IgG2a, κ), MCAM (FITC antihuman CD146, clone P1H12, isotype: Mouse IgG1, κ). For viability, an Annexin-V-pacific blue conjugate (Life-technologies, Eugene, OR, USA) and PI were used. As an isotype control, the following antibodies were used: PE clone MOPC-173 mouse IgG2a, κ; PE/Dazzle594, clone RTK2071, isotype: Rat IgG1, κ; BV711 clone IM7, Rat IgG2b, κ; AF647 clone RTK2758, isotype Rat IgG2a, κ; FITC clone MOPC-21, mouse IgG1, κ. All single and mixed (A-V + PI and CD44 + MCAM + Vimentin) PCRs were performed according to the manufacturer protocol without any modifications. Samples were analyzed with an ID7000 Sony Spectral Flow cytometer (Sony Biotechnology, San Jose, CA, USA). The ID7000 used in this study was equipped with four lasers 405/488/561/637 nm, and PMT gains/voltages were independently adjusted for each laser. For each sample, a total of 10^4^ cells were analyzed. Before analysis, the Sony ID7000 was calibrated using alignment checks (Sony Biotechnology Inc. AlignCheck Flow Cytometer Alignment Beads 10^7^/mL 10 μm, 2 mL, cat. no AE700510) and the eight-peak performance beads (Sony Biotechnology Inc. Eight Peak Bead cat. no AE700522, 10^7^/mL 3.1 μm, 5 mL cat. no AE700510), following the instrument supplier’s guidelines. Initial gating was established using unstained and isotype control samples to assess background signal and nonspecific binding. Minimal background staining was detected across all fluorochrome channels, with nonspecific events remaining below 0.2%. Based on these controls, a fluorescence threshold corresponding to 10̂4 signal intensity units was applied uniformly across samples to distinguish between negative and positive populations (Supporting Information Figures S1 and S2).

Gates for positive signal detection on spectral plots were initially positioned automatically by the analysis software. These gates, indicated by black dashed lines, were aligned with the expected emission spectra of each fluorochrome. The default gate placement was reviewed and adjusted manually when necessary, based on fluorescence intensities observed in the unstained and isotype control samples, to ensure accurate discrimination and reproducibility across experimental conditions.

### 2.8 Statistical and Protein Interactions Analysis

GraphPad Prism v.8 Software (GraphPad Software, San Diego, CA, USA) was used for statistical interpretation of obtained data. Results are presented as means with standard deviation, with *p* values corresponding to (*) *p* ≤ 0.05, (**) *p* ≤ 0.01, (***) *p* ≤ 0.001, and (****) *p* ≤ 0.0001.

For prediction of common protein interactions, two proteins were analyzed by means of a Search Tool for the Retrieval of Interacting Genes/Proteins (STRING).^16^

## 3 Results

### 3.1 Evaluation of Growth and Morphology Changes in Primary and Metastatic Melanoma

Significant differences in FM55p tumorspheres structure and growth were observed since the 14th day of experiment (Figure 2A–C and Table S2, Supporting Information). Exceptionally big-sized FM55p tumorspheres after 28 days reached the unique diameter of ∼2 mm (2000 ± 65 μm) and a perimeter exceeding 7 mm. Interestingly, control spheroids exhibited structural disintegration due to widespread cell death, as revealed by living cell counting (Figure 2D). Doubling-time (DT) calculations based on living cells population and tumorspheres volume^17^ showed that those cultured in a bioreactor had significantly (*p* = 0.004 and *p* = 0.0007 respectively, Figure 2E) reduced DT when compared to the control.

**Figure 2:**
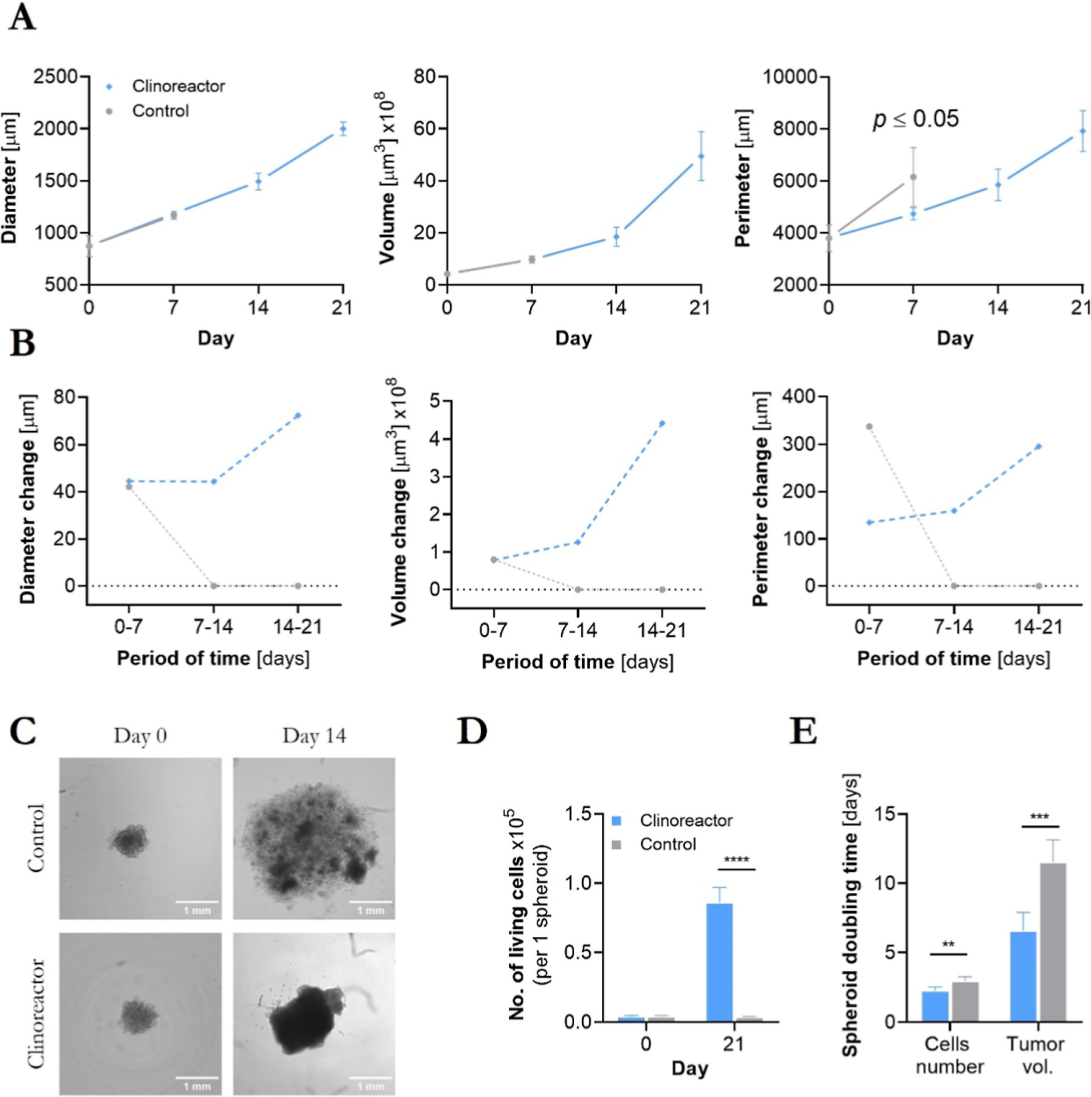
Primary melanoma (FM55p) tumorspheres growth. Morphological changes (A) and tumor growth rates differences between the control and tested groups. (B) Due to the destruction of control spheroids, data presented in part A do not contain relevant measurements for reference spheroids; example of BF images used for analysis with imaging cytometry (C); distribution of living cells in tumorspheres (D); spheroid doubling time calculations based on single-cell analysis and tumor volume (E).

As shown in Figure 3, no disintegration or spheroid death was observed for metastatic melanoma models. However, significant differences were noticed in growth and morphology starting from the first week after inoculation in a bioreactor (Figure 3A–C and Table S3 Supporting Information). Tumorspheres reached an average diameter of 1432 ± 54 μm, approximately 33% larger than the control. Enhanced growth was associated with significantly (*p* ≤ 0.0001) increased number of living cells found in bioreactor-based tumorspheres and 25–50% shortened doubling time depending on cell number or tumor volume calculations (Figure 3D,E).

**Figure 3:**
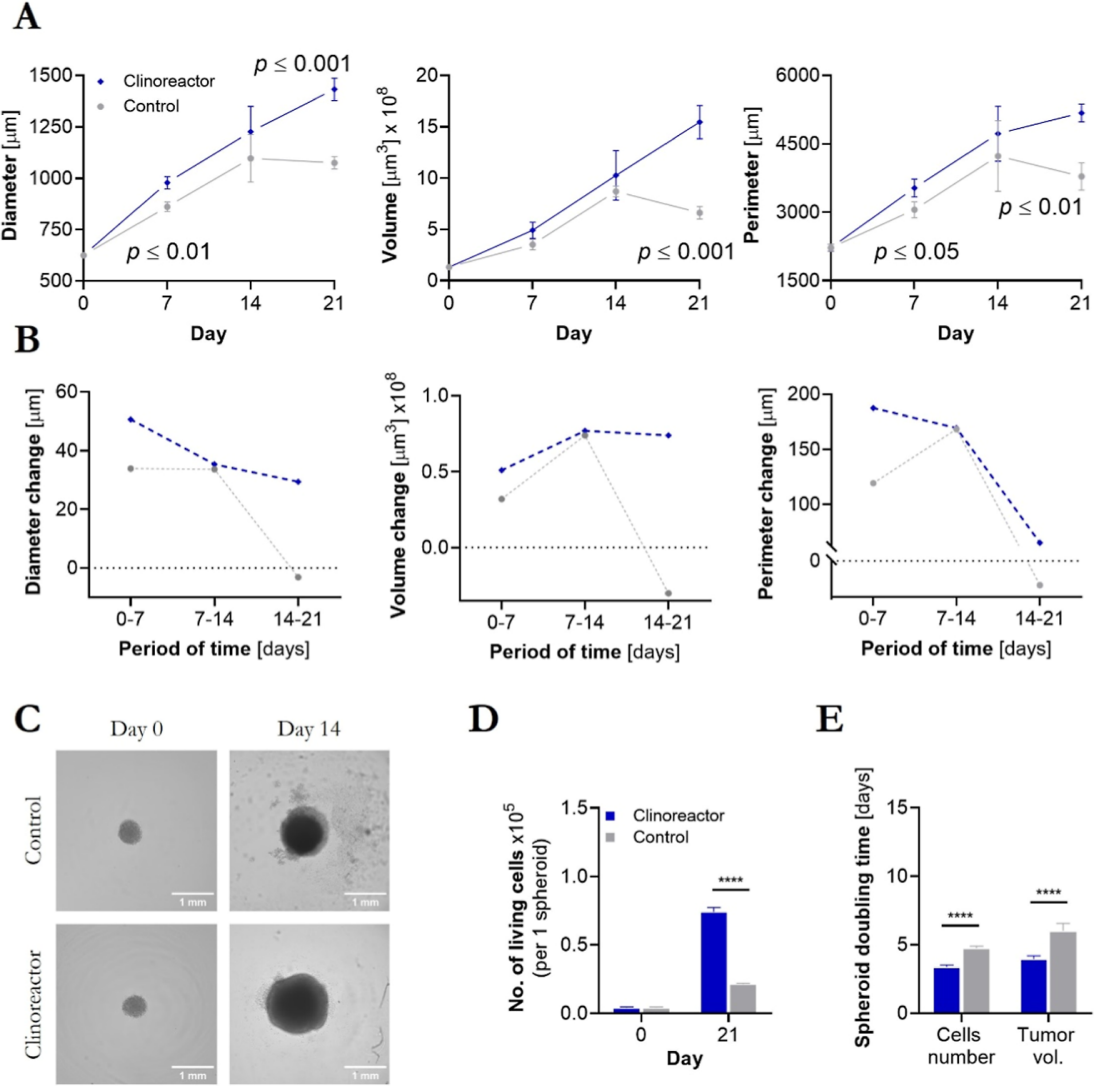
Metastatic melanoma (WM266-4) tumorspheres growth. Morphological changes (A) and tumor growth rate differences between the control and tested group (B); example of BF images used for analysis with imaging cytometry (C); distribution of living cells in tumorspheres (D); spheroid doubling time calculations based on single-cell analysis and tumor volume (E).

### 3.2 In-Depth Tumorspheres Analysis

Remarkably expressed morphological differences between tested methods inspired us to verify how the external changes link to the tumor inside. To date, in-depth *in vitro* 3D tumor assessment is rarely executed, mostly due to numerous challenges of such examination.^18^ Confocal BF visualization of tumor cross sections as well as fluorescent imaging of whole tumors (Figure 4) were performed at day 14 when FM55p control spheroids’ disintegration was noticed, and the aim of this imaging was to identify possible reasons for this phenomenon.

**Figure 4:**
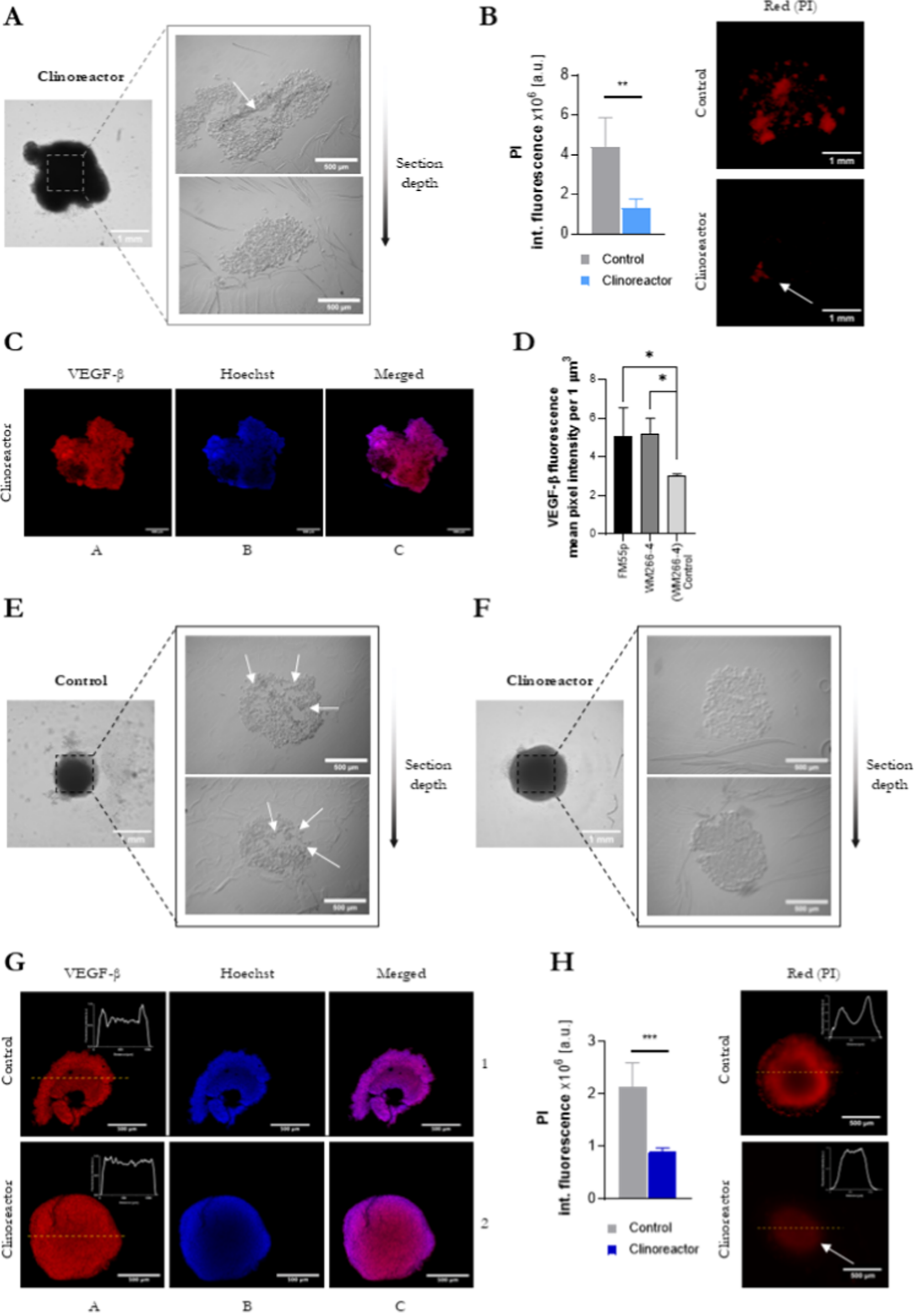
In-depth tumorspheres visualization. Parts A–C correspond to FM55p and I–H to WM266-4. BF tumorspheres imaging followed by confocal visualization of top and middle part cross sections (A,E,F). Tumorspheres from FM55p cells were not solid, become deformed during sectioning (A), and contained empty spaces inside (white arrow). Spheroids cultured in the control condition disintegrated after 7 days (picture not shown). Spheroids and tumorspheres from the WM266-4 cell line were more stable, but the empty space in the control condition had been formed (white arrows) (E), not visible in Clinoreactor tumorspheres (F). In FM55p cells, necrotic zones were spread throughout the entire spheroid, the size of which was much larger in the control spheroids than in the Clinoreactor tumorspheres. (B) High PI fluorescence intensity was observed in control conditions. In WM266-4 cells, PI intensity revealed lot of necrotic cells in a control speheroid (H), and PI distribution showed a mild necrotic zone in Cinoreactor tumorspheres. The scale bar in images (C) and (G) corresponds to 500 μm.

The internal structure of tumorspheres from the bioreactor was compact with high cell density and only a single stripping of cells areas (Figure 4A,F, marked with white arrows). We did not identify any changes in compactness with increasing section depth.

Despite the satisfactory external morphology of the WM266-4 control group as well, we found there much more structural diversities, e.g., empty spaces and looser cell-cell connections (Figure 4E). These observations were in line with necrotic core staining that revealed increased dead cell content located internally. In particular, the PI signal from necrotic cells in both (FM55p and WM266-4) control spheroids was intense and widespread among tumors (Figure 4B,H). An increase in PI fluorescence was found in larger clusters of organized cells, but PI-positive cells were also detected in the area of single cell layers. Conversely, tumorspheres from the bioreactor revealed only trace PI + cells content (*p* = 0.002 and *p* = 0.0004 for FM55p and WM266-4, respectively) localized mostly in the form of small foci in the midsection. Quantitative analysis of the PI distribution in WM266-4 cells showed that in the inner zone of the Clinoreactor tumorsphere, the number of necrotic cells is lower than in a control spheroid (H).

In both lines, VEGF-β expression (C,G) was observed, but a WM266-4 pattern showed a higher expression of VEGF-β in Clinoreactor tumorspheres (D).

Subsequently, we visualized the expression of VEGF-β (Figure 4C,G). VEGF-β is one of the major hallmarks describing the development of the tumor tissue, providing information about the capability for vascularization, crucial in predicting tumor growth.^19,20^ The 3D *z*-stack-reconstructed images revealed that externally located cells show a high expression of VEGF-β both in the Clinoreactor and the plate-based control. Quantitative analysis showed that the signal intensity in tumorspheres was significantly (*p* ≤ 0.05) higher and equally distributed when compared to the control (4G).

### 3.3 Protein Expression and Epithelial-to-Mesenchymal Transition

Distribution analysis of FM55p living cells evaluated with Annexin-V and PI single-cell staining showed a notably higher A-V (+) and PI (+) cell content (Figure 5A,C) when compared to the tumorspheres from bioreactors (Figure 5B,C), especially at day 14. In general, the living cells content assessed with flow cytometry was in line with trypan-blue exclusion staining, indicating an elevated number of dead cells in control spheroids. The increase of PI + cell number in tumorspheres at day 21 is probably an aftermath of larger tumor size when compared to day 14; however as we showed previously, it did not impair or inhibit tumorsphere growth (Figure 2A,B). Protein expression analyses performed with reference to the level of respective proteins in 2D cell cultures (Figure 5D) showed low levels of E-cadherin and VE-cadherin (<2.5%), and hence did not justify quantitative analysis of epithelial-to-mesenchymal transition (EMT), which is one of the major hallmarks of cancer progression and metastatic sites formation. Interestingly, significant alterations in proteins expression were found in all of the other markers. Progressing over time, changes in the expression of MCAM and CD44 in tumorspheres were identified mostly at day 21 (*p* = 0.0005 for MCAM and *p* = 0.0010 for CD44) while the vimentin level was downregulated to 3.6 ± 1.6% at day 21, probably due to suppressing the EMT^21^ (Figure 5E).

**Figure 5:**
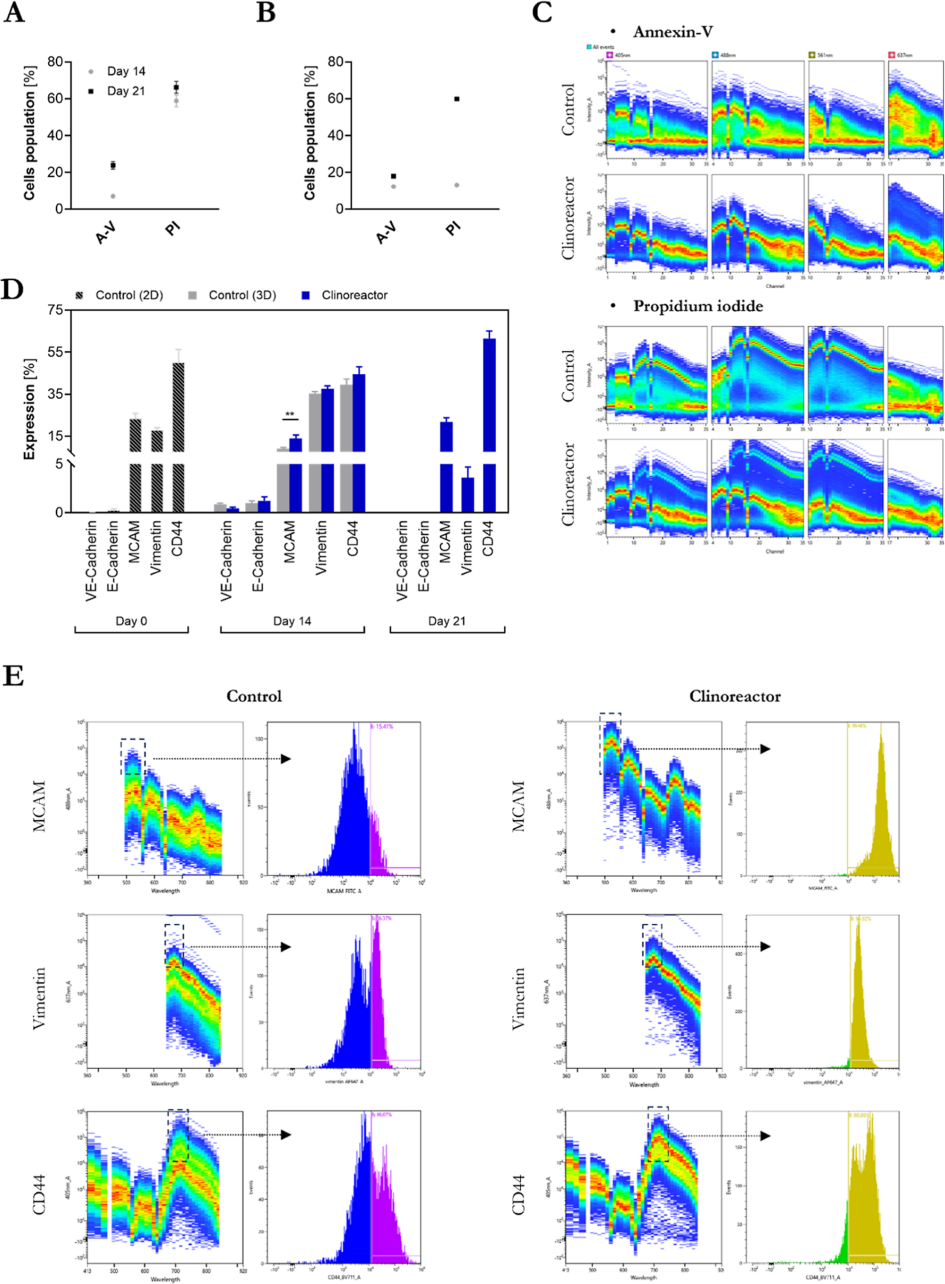
Spectral flow cytometry single-cell analyses of FM55p cells. Single -cell viability test (A–C): percentage of Annexin-V (A-V)- and PI-stained cells in (A) 3D control spheroids cultured in 96-well plates and bioreactor-grown tumorspheres (B) at days 14 and 21 and representative spectral flow cytometry plots (C). Colored density plots represent individual cell fluorescence intensities. Modulators of the EMT and angiogenesis (D,E): quantification of the expression of the VE cadherin, E cadherin, MCAM, Vimentin, and CD44 in 2D monolayer cultures (day 0) and spheroids (day 14 and day 21) (D). Bars show the percentage of marker-positive cells. Note: due to disintegration of 3D control spheroids, no data are available for control samples at day 21. (E) Representative spectral flow cytometry plots for MCAM, Vimentin, and CD44 markers at day 14 of spheroid growth. Left panels show single-cell density plots with signal distributions, while right panels show gated histograms highlighting positive cell populations (indicated by black arrows). Gating regions are marked with black boxes. Gates were generated automatically from spectral signatures using the cytometer software. A fluorescence intensity threshold of 10^4^ was used to distinguish a positive-cell population.

The number of living cells in WM266-4 tumorspheres was significantly higher when compared not only to the control but also in reference to FM55p (Figure 6A,C). Conversely to the primary melanoma, WM266-4 showed a reduced PI signal, especially at day 21 when FM55p and WM266-4 control revealed an increase of necrotic cells that progressed over time. Based on protein expression analyses, levels of MCAM, vimentin, and CD44 expression were significantly elevated when compared to the control. Similarly to previously observed changes, WM266-4 tumorspheres also downregulated vimentin expression during the last week of growth, but interestingly, also slight reduction of CD44 level was noted (Figure 6D,E).

**Figure 6:**
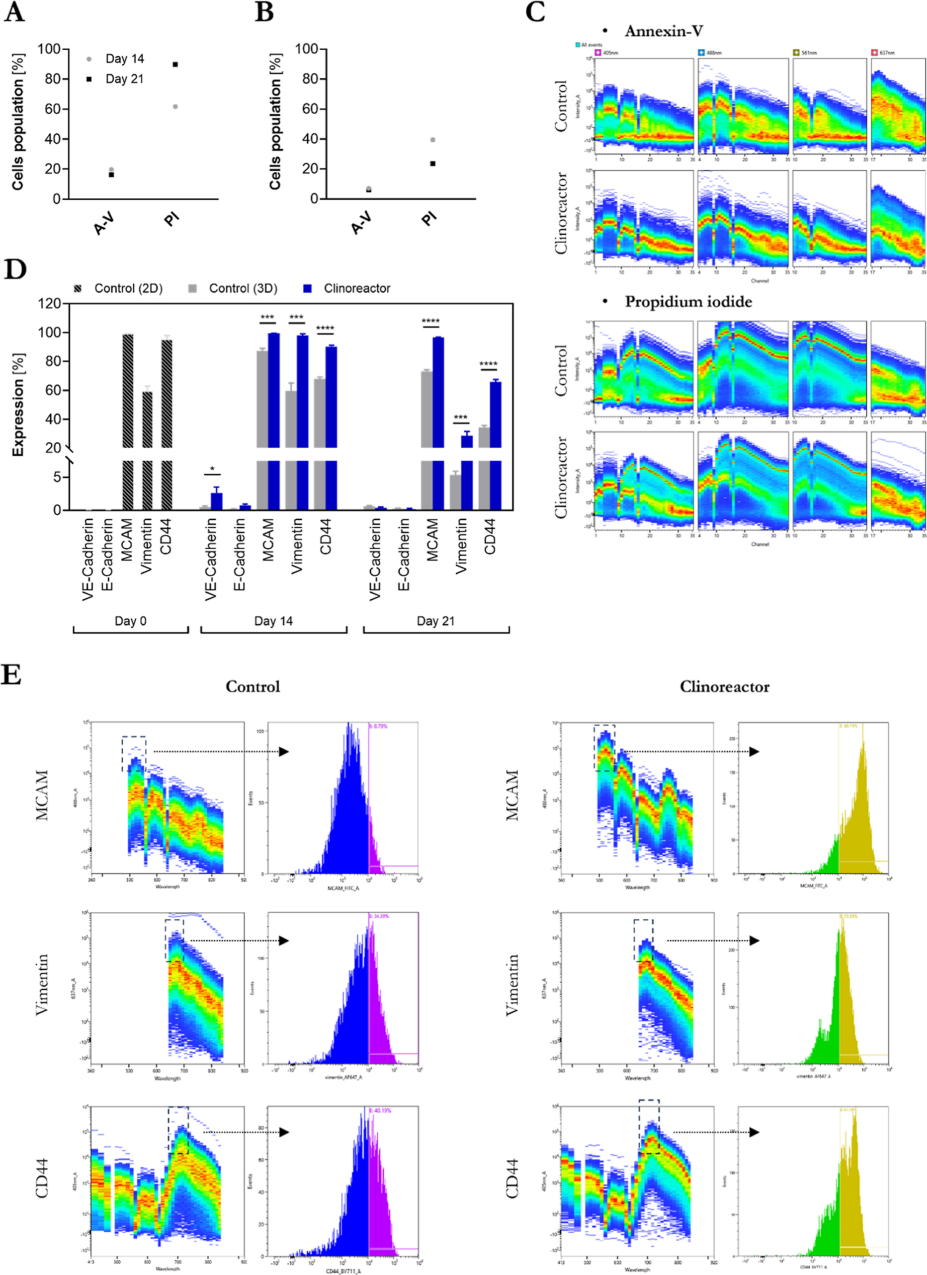
Spectral flow cytometry single-cell analyses of WM266-4 cells. Single-cell viability test (A–C): percentage of Annexin-V (A-V)- and PI-positive-stained cells’ distribution in (A) 3D control spheroids cultured in 96-well plates and bioreactor-grown tumorspheres (B) at days 14 and 21 and representative spectral flow cytometry plots (C). Colored density plots represent individual cell fluorescence intensities. Modulators of the EMT and angiogenesis (D,E): quantification of the expression of the VE cadherin, E cadherin, MCAM, Vimentin, and CD44 in 2D monolayer cultures (day 0) and spheroids (day 14 and day 21) (D). Quantification of the expression of the VE cadherin, E cadherin, MCAM, Vimentin, and CD44 in 2D monolayer cultures (day 0) and spheroids (day 14 and day 21). Bars show the percentage of marker-positive cells. (E) Representative spectral flow cytometry plots for MCAM, Vimentin, and CD44 markers at day 14 of spheroid growth. Left panels show single-cell density plots with signal distributions, while right panels show gated histograms highlighting positive cell populations (indicated by black arrows). Gating regions are marked with black boxes. Gates were generated automatically from spectral signatures using the cytometer software. A fluorescence intensity threshold of 10^4^ was used to distinguish a positive-cell population.

### 3.4 Protein Coexpression and STRING Analysis

Analysis of proteome changes additionally allows for understating the interactions of cellular metabolic pathways and their implication in *in vitro* tumor modeling, especially when their up- or downregulation is associated with other molecular markers. For this purpose, we first examined the CD44, MCAM, and vimentin simultaneous expression in both of the tested melanomas. As shown in Figure 7A, both FM55p and WM266-4 tumorspheres cultured in a bioreactor showed overall significantly higher proteins’ coexpression than control groups, regardless of the markers being considered. Moreover, plate-based spheroids did not differ significantly between each other (*p* = 0.8629), ultimately showing limitations and disadvantages of the traditional model. Interestingly, statistical analyses revealed that in primary melanoma, simultaneous expression occurs more frequently when compared to the metastatic models (88% vs 60%) but any specific correlation was not identified among tested subgroups including CD44 + vimentin, CD44 + MCAM, and vimentin + MCAM.

**Figure 7:**
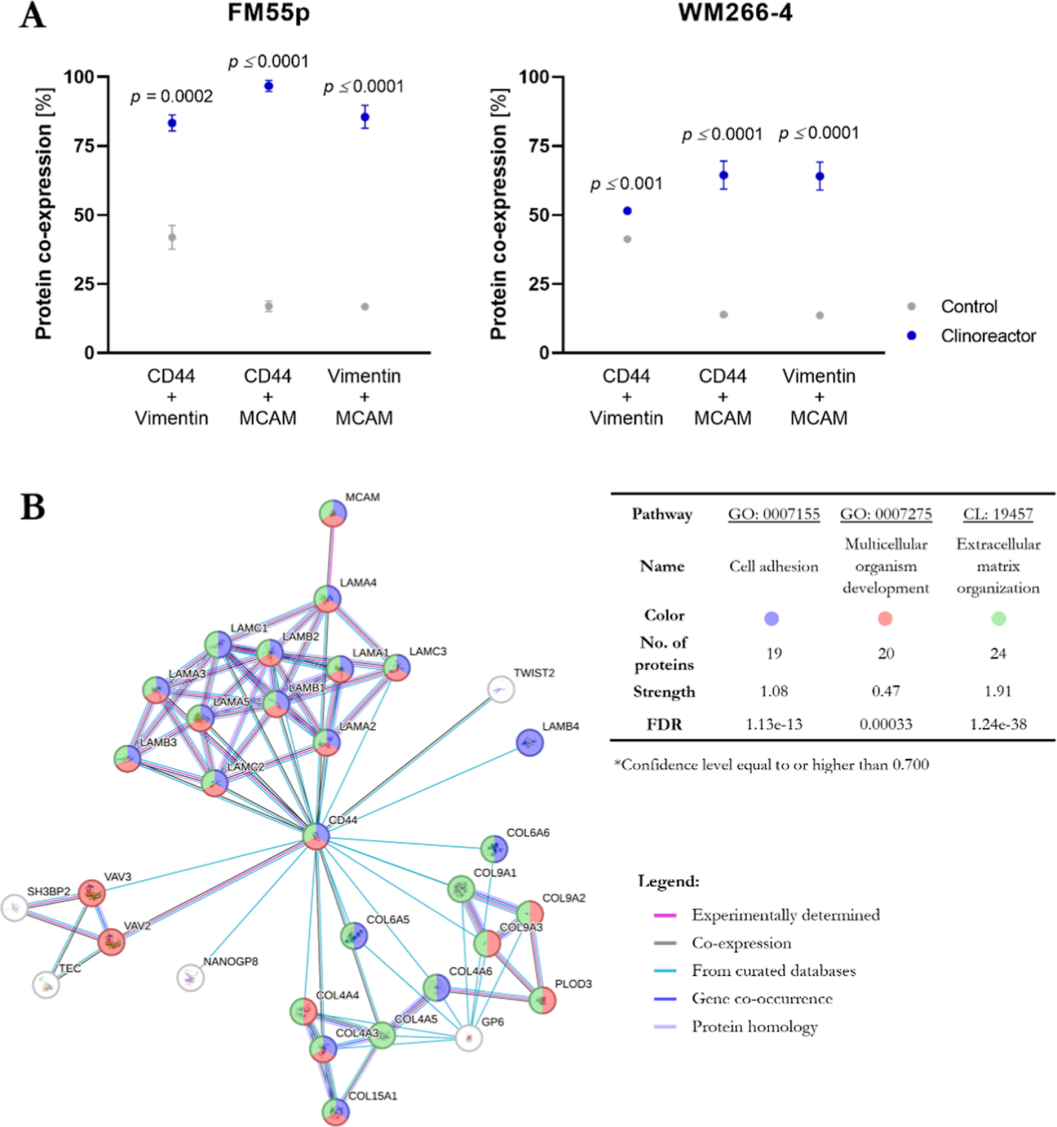
CD44, vimentin, and MCAM simultaneous expression in FM55p and WM266-4 obtained structures—simultaneous expression of CD44/vimentin/MCAM was assessed with mixed (triple) staining and analyzed with spectral flow cytometry (A); STRING analysis of the protein–protein interaction network based on identified simultaneous expression—confidence level equal to or higher than 0.700 ^16^ (B).

STRING analysis (Figure 7B) of coexpressed proteins revealed that dual upregulation of CD44 and MCAM is frequently associated with various interactions mediated by laminins (LAMA1-5; LAMB1-3; LAMC1-3) and collagens (COL4A3 and COL15A1). Conversely, any correlation with vimentin as a third of coexpressed protein was not found. Therefore, based on the protein profile of analyzed tumorspheres, it can be concluded that a higher development of single cells translates to a higher maturity of the entire system.

## 4 Discussion

One of the most significant potential applications of the presented model is in drug screening and preclinical evaluation. We used in our study, two melanoma cell lines, since the malignant WM266-4 line carries a BRAF p.V600E substitution—missense mutation (ENST00000288602.11).^22^ We compared spheroids’ and tumorspheres’ morphology and preclinically designed end points such as viability tests (PI imaging of necrotic core, PI/Annexin V flow cytometry, trypan blue staining). We also tested the modulators of the EMT pathway (spectral flow cytometry of vimentin and MCAM and CD44 expression) to figure out differences between BRAF and non-BRAF positive melanoma cell lines using large tumorspheres.

Implementing a bioreactor offers several technological advantages, stating that suspending the cells in a static orbit during rotation provides a protective function against shear stress. Increased access to nutrients and oxygen ensures a better supply for the vital elements, while effective waste elimination prevents undesirable cell poisoning because of the accumulation of metabolic products. Consequently, strengthening of cell-to-cell interactions is observed, and advanced model development can be achieved. Discussed morphological and physiological changes play an important role in the intact expression of proteins and genes; thus evolving TME leads to achieving more clinical-like tumor models.^23,24^

The obtained tumorspheres showed an unprecedented size and, more importantly, preserved the proliferation capacity and biochemical functions during a month-long experiment on a population of 1100 tumorspheres originated from primary and metastatic melanoma. While the diameter of most commonly used spheroids is in the range of 250–700 μm^25^ and usually does not reach 1000 μm,^26^ in our study, we obtained large 3D tumorspheres with an over 2 mm (2.3 mm maximum) diameter corresponding to 5.0 ± 0.9 mm^3^ of the estimated volume, which was previously impossible *in vitro*.^27,28^ This approach can be used for testing the response to a BRAF-targeted therapy in personalized medicine or drug resistance studies to replace animal xenograft studies to prevent drug resistance.^29^ However, our model needs to be evaluated using BRAF/MEK inhibitors (e.g., vemurafenib) to help validate the functional fidelity of this model compared to 2D.

Given the size of the spheroid and the prolonged culture, we expect hypoxic gradients to develop in the tumorspheres. In further studies, we plan to investigate markers such as HIF-1α or carbonic anhydrase IX, which are typical indicators of hypoxic microenvironments and are important for the biological fidelity of the TME, and to correlate these hypoxia markers with the new biomarker positronium.^30^

In our study, we observed a low expression of E-cadherin and VE-cadherin, which unfortunately limited our analysis of EMT. There are alternative markers of this process, such as *N*-cadherin, ZEB1, SNAI1, or TWIST1, which could be more useful. ZEB1 is a major regulator of melanoma cell plasticity, driving resistance to mitogen-activated protein kinase-targeted therapies. This transcription factor is a key determinant of melanoma immune escape, and its targeting may increase the efficacy of immunotherapy in melanoma, which is still not tested in a tumorsphere model.^31^

Apart from the morphological maturity that was enabled due to a higher cell viability, the TME was found to be at a higher development level as well. Increased VEGF-β expression proved that the vascularization capacity is enhanced; thus *in vivo* angiogenesis shows great prospects for undergoing efficient modulation with the discussed models.^32^ Coexpression of MCAM and CD44 found in over 95% (FM55p) and 65% (WM266-4) of cells is indirect but it is solid evidence that tumorspheres from the bioreactor can also restore or acquire the tissue-like functions. Our predictions for CD44 and MCAM proteins revealed the involvement of two extracellular matrix (ECM) protein pathways: the laminin 332 gene family and type IV collagen alpha chain (COL4A). LM 332 (formerly called LM 5), as a specific variant of laminin glycoprotein, is a heterotrimer of α3, β3, and γ2 chains, encoded by LAMA3, LAMB3, and LAMC2, respectively. LM332 forms the epithelial-basement membrane (BM) and supports the main function of epithelial tissue such as formation, healing, and regeneration. LM332 is also considered a hallmark of cancer development, and its expression is impaired in various cancer cells (invasive mammary, colon, melanoma, and sarcoma).^33^ COL4A is also a major component of BM, and its role in tumor angiogenesis and progression has been widely investigated.^34^

Overexpression of these two ECM components was observed in our study as strong intercellular interactions in the tested spheroids, which made cell separation difficult (it was necessary to use accutase to digest the spheroids) and constituted a mechanical barrier for dyes and antibodies used in the visualization of spheroids. Similar problems regarding the Ab-based detection in 3D models were previously reported by Mitrakas;^35^ thus it was not possible to detect VEGF-β deeper in whole tumorsphere structure, enabling only boundary Ab penetration.

In general, the discussed approach can be easily and repeatedly used for other cellular models. Low SDs with a highly representative tumorsphere population were observed not only within the experimental groups but more importantly between separate experimental runs. Due to high morphological and physiological development, the presented models can serve not only as convenient in further *in vitro* research but principally as a standalone biological model that may provide complementary data at various stages of the drug development process. Possible implications of the presented *in vitro* models may primarily fill the gap in biological examination in the preclinical stage but also show great potency to be applicable for analysis with clinically used diagnostic systems. This includes particular molecular imaging specifically for research into the development of new radiopharmaceuticals and multiphoton imaging in novel positron emission tomography and single photon emission computed tomography devices and may provide a completely new view of the field, regarding intratumoral markers expression, heterogeneity, and metabolism based on currently developed approaches.^36,37^ The *in vitro* models available to date do not support such an interdisciplinary examination due to numerous features.

The best known reference for a drug rest in melanoma therapy is the BRAF/MEK pathway inhibitor vemurafenib, which is still the most effective drug for patients carrying this mutation. Since almost half of these patients do not show a response to therapy and a quarter of those who have regression develop drug resistance, personalizing therapy is the biggest challenge in melanoma patients. Using our tumorsphere model is the main goal of our future research on personalized treatment.

Our findings presented in this article have proven that it is possible to design and develop remarkable biological models that can bridge the gap between *in vitro* and *in vivo* systems and become the basis of future cancer research. The results showed important characteristics of spheroid properties obtained for the first time in the millimeter-range size, with retained growth ability over several weeks of culture and most importantly highly established TME. Expanding of 3D spheroids or organoids themselves is a major strategy used in the study of cellular components. The presented results show great prospects to develop hybrid 3D cellular systems combining the features of spheroids and organoids for preclinical research.

## Supplementary Material



## Notes

Additional methodologies and data, including mathematical formulas of spheroids’ doubling time calculations, shape parameters and units, and spectral flow cytometry isotype control setting (PDF)
