# Supplementary material for: Developing a Tumor Microenvironment in Rotating Human Melanoma Cell Cultures: Study of a Novel Preclinical Model
Source: ACS Omega. 2025 Jun 20;10(25):27288–300. doi: 10.1021/acsomega.5c02682 (PMC12223851; doi:10.1021/acsomega.5c02682)
Supplement: Supplementary file 1 [file ao5c02682_si_001.pdf]

Developing tumor microenvironment in rotating human melanoma cell cultures: study of novel preclinical model

Kamil Wawrowicz[a,b], Martyna Durak Kozica[a,b], Mateusz Wierzbicki[c], Ewa Ł. Stępień[a,b *]

a Department of Medical Physics, M. Smoluchowski Institute of Physics, Faculty of Physics, Astronomy and Applied Computer Science, Jagiellonian University, Kraków, Poland
b Center for Theranostics, Jagiellonian University, Kraków, Poland
c Department of Nanobiotechnology, Institute of Biology, Warsaw University of Life Sciences, Warsaw, Poland

SUPPLEMENTARY INFORMATION

Eq S1. Mathematical formulas used for calculations spheroids doubling time.

Cell number:

$$DT = \frac{T \cdot ln(2)}{ln(\frac{N_b}{N_a})};$$

where: T – time; N_b – measured cell number after time T; N_a – starting cell number

Tumor volume:

$$DT = \frac{T \cdot ln(2)}{ln(\frac{V_b}{V_a})};$$

where: T – time; V_b – measured tumor volume after time T; N_a – starting tumor volume

Table S1. Mathematical formulas used for tumorspheres morphology assessment.

Parameter	Formula
Area (*A*)	Pixels number detected inside the identified spheroid
Diameter	A= π·[radius]^2
Perimeter	Length of the tumorsphere edge
Est. volume (*V*)	V= (π/6)·[short diam.]^2·[long diam.]*
Shape factor (circularity)	Tumorspheres breadth to the length**
Form factor (*FF*)	Perimeter: area ratio**

* Mathematical modelling assuming that spheroid thickness is equal to the shortest measured diameter. ** unitless

Table S2. Shape and form factors of FM55p spheroids.

		Day 0	Day 7	Day 14	Day 21
**Shape factor**	Control	0.25 ± 0.06	0.16 ± 0.07	*No data*	*No data*
	ClinoReactor	0.25 ± 0.06	0.38 ± 0.06	0.36 ± 0.06	0.32 ± 0.05
**Form factor**	Control	0.55 ± 0.13	0.38 ± 0.12	*No data*	*No data*
	ClinoReactor	0.55 ± 0.13	0.62 ± 0.04	0.65 ± 0.08	0.62 ± 0.10

Table S3. Shape and form factors of WM266-4 spheroids.

		Day 0	Day 7	Day 14	Day 21
**Shape factor**	Control	0.64 ± 0.12	0.55 ± 0.05	0.41 ± 0.15	0.60 ± 0.02
	ClinoReactor	0.64 ± 0.12	0.68 ± 0.23	0.51 ± 0.09	0.80 ± 0.11
**Form factor**	Control	0.78 ± 0.04	0.79 ± 0.05	0.54 ± 0.15	0.81 ± 0.08
	ClinoReactor	0.78 ± 0.04	0.78 ± 0.05	0.70 ± 0.04	0.77 ± 0.01

Figure S1. Spectral flow cytometry – isotype control FM55p cells

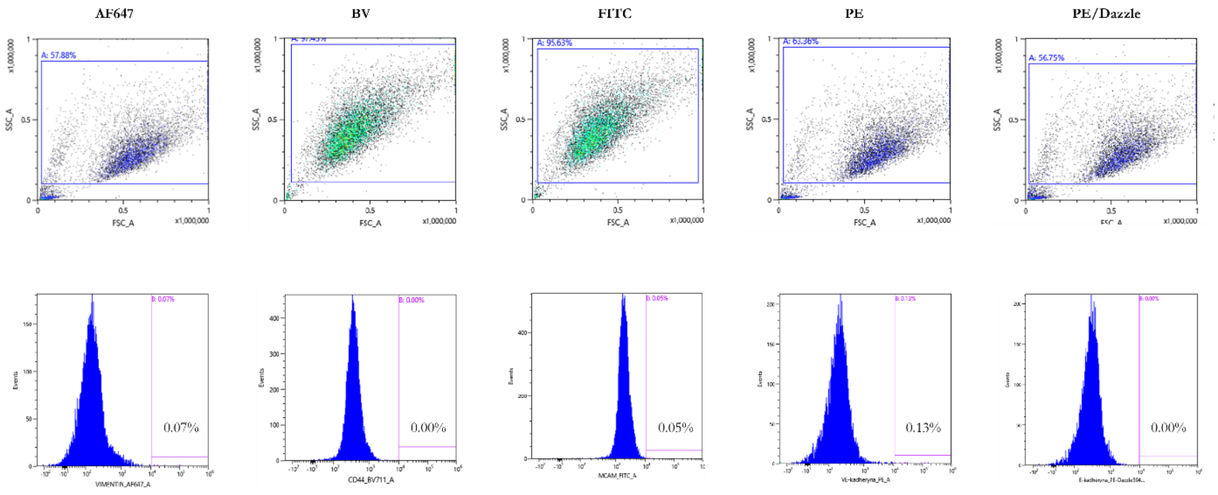

Representative dot plots (top row) and corresponding histograms (bottom row) showing fluorescence profiles of FM55p cells stained with isotype control antibodies conjugated to AF647, BV, FITC, PE, and PE/Dazzle fluorochromes.

The dot plots illustrate the scatter characteristics (FSC-A vs SSC-A), and the histograms display the fluorescence intensity distributions for each fluorochrome channel.

Minimal background staining was observed across all channels, with positive events remaining below 0.2%.

The fluorescence threshold corresponding to 10^4 signal intensity, as determined from these isotype controls, was applied during the gating strategy for the analysis of stained experimental samples to ensure consistent discrimination between negative and positive populations.

Figure S2. Spectral flow cytometry – isotype control WM266-4 cells

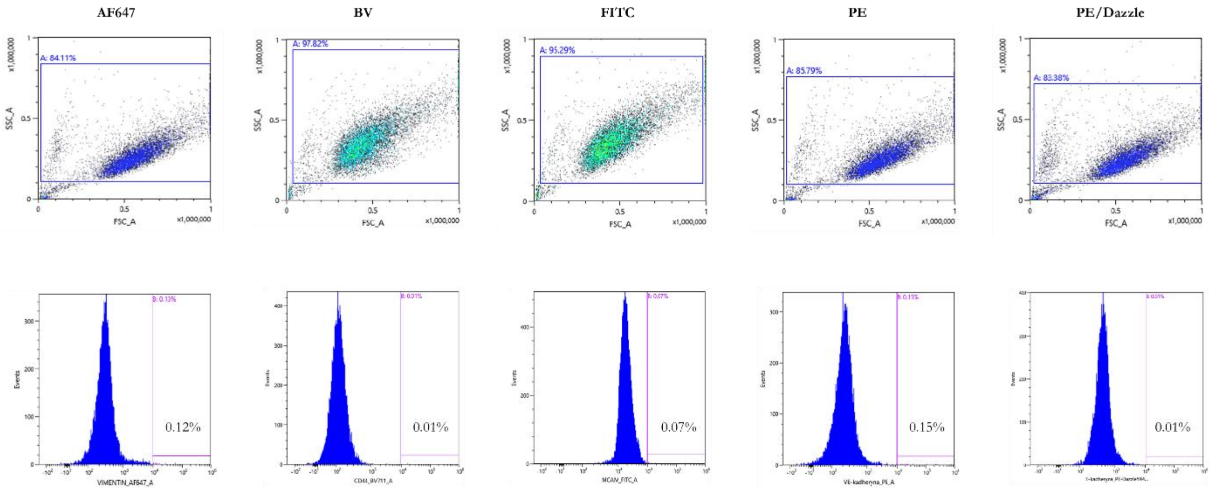

Representative dot plots (top row) and corresponding histograms (bottom row) showing fluorescence profiles of WM266-4 cells stained with isotype control antibodies conjugated to AF647, BV, FITC, PE, and PE/Dazzle fluorochromes.

The dot plots illustrate the scatter characteristics (FSC-A vs SSC-A), and the histograms display the fluorescence intensity distributions for each fluorochrome channel.

Minimal background staining was observed across all channels, with positive events remaining below 0.2%.

The fluorescence threshold corresponding to 10^4 signal intensity, as determined from these isotype controls, was applied during the gating strategy for the analysis of stained experimental samples to ensure consistent discrimination between negative and positive populations.
